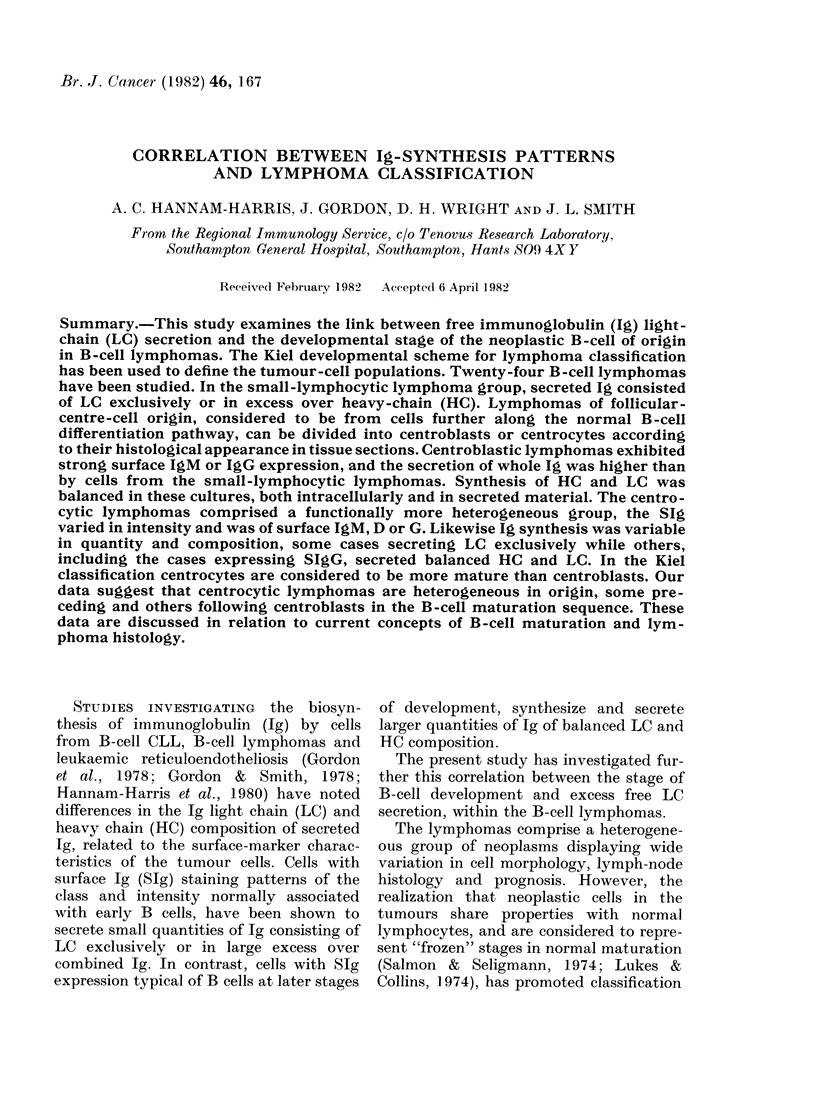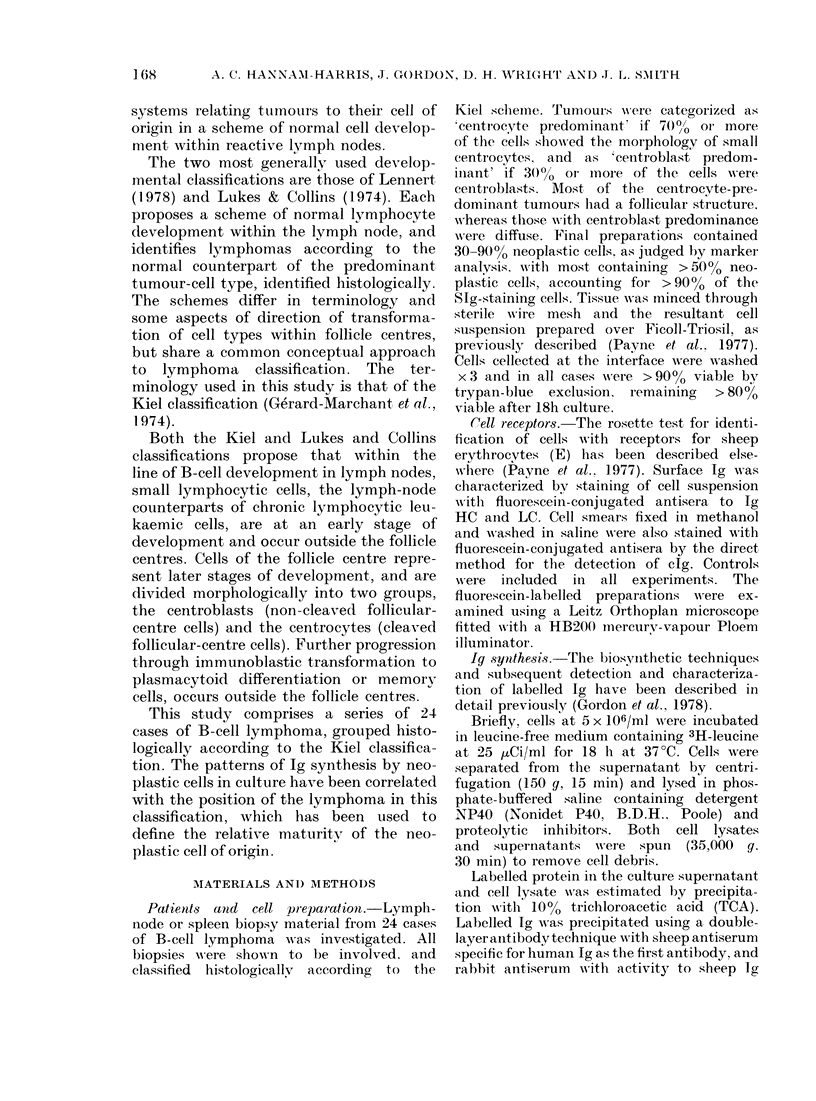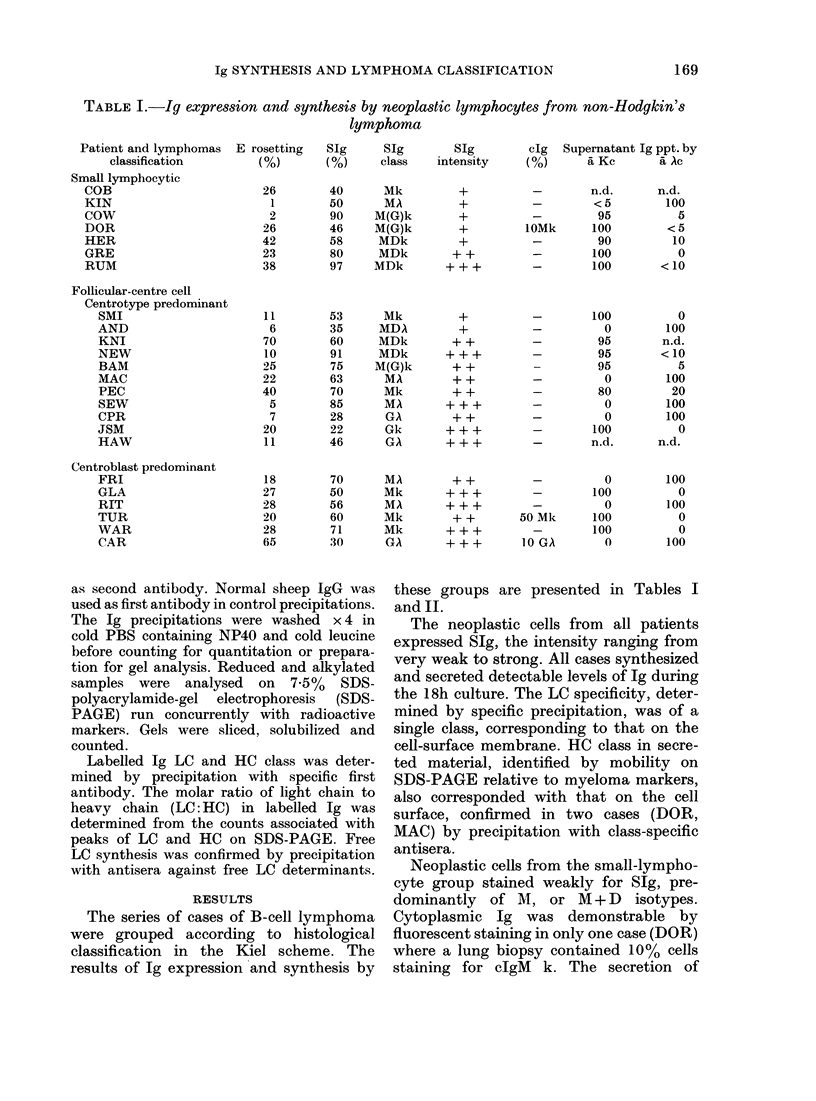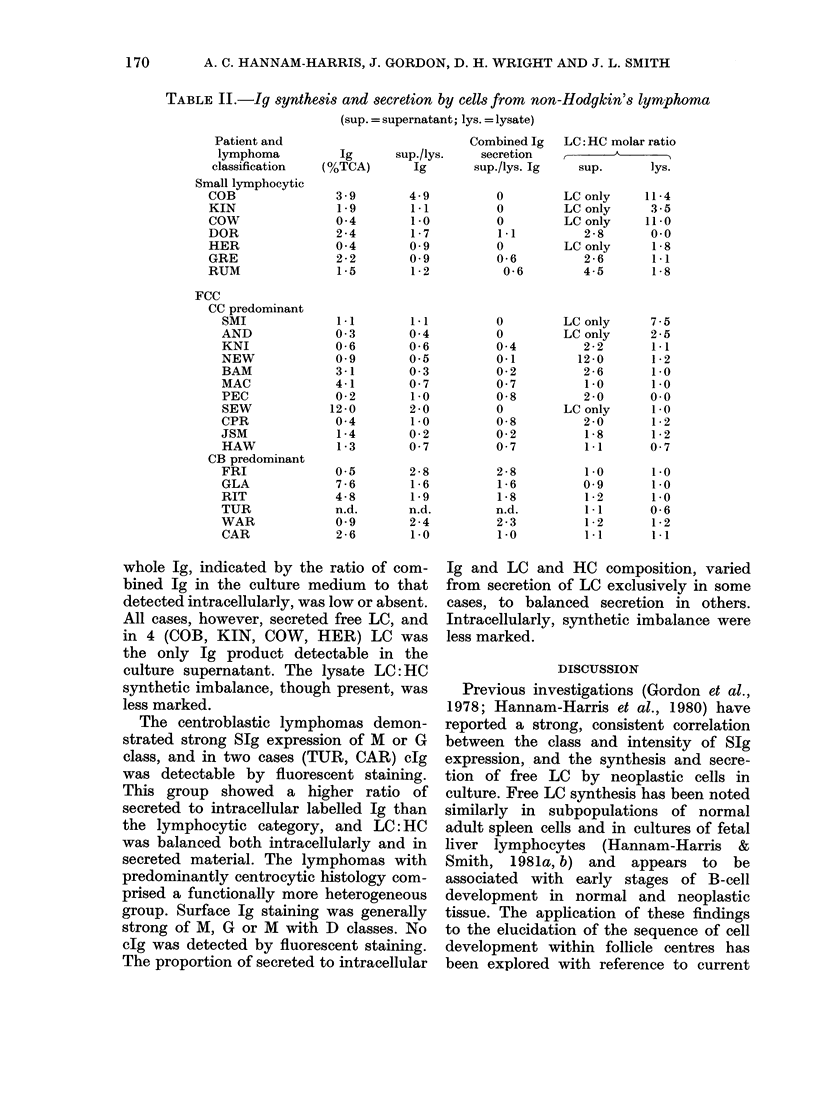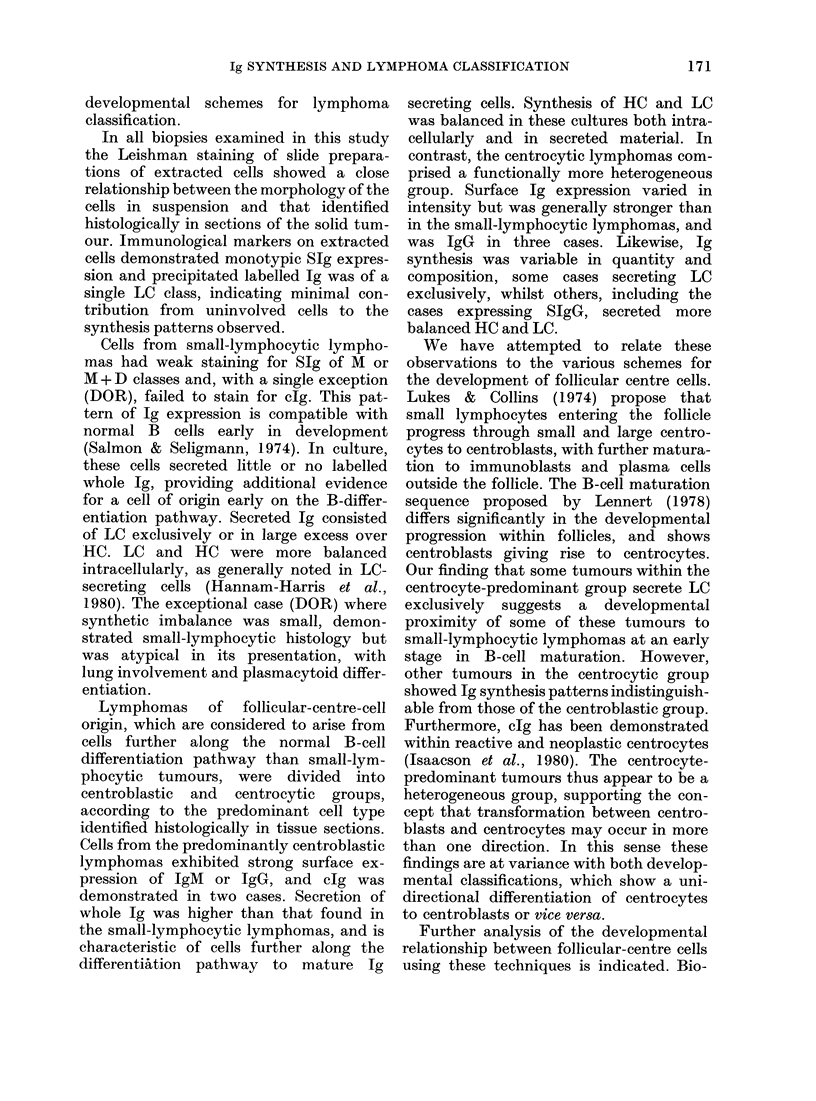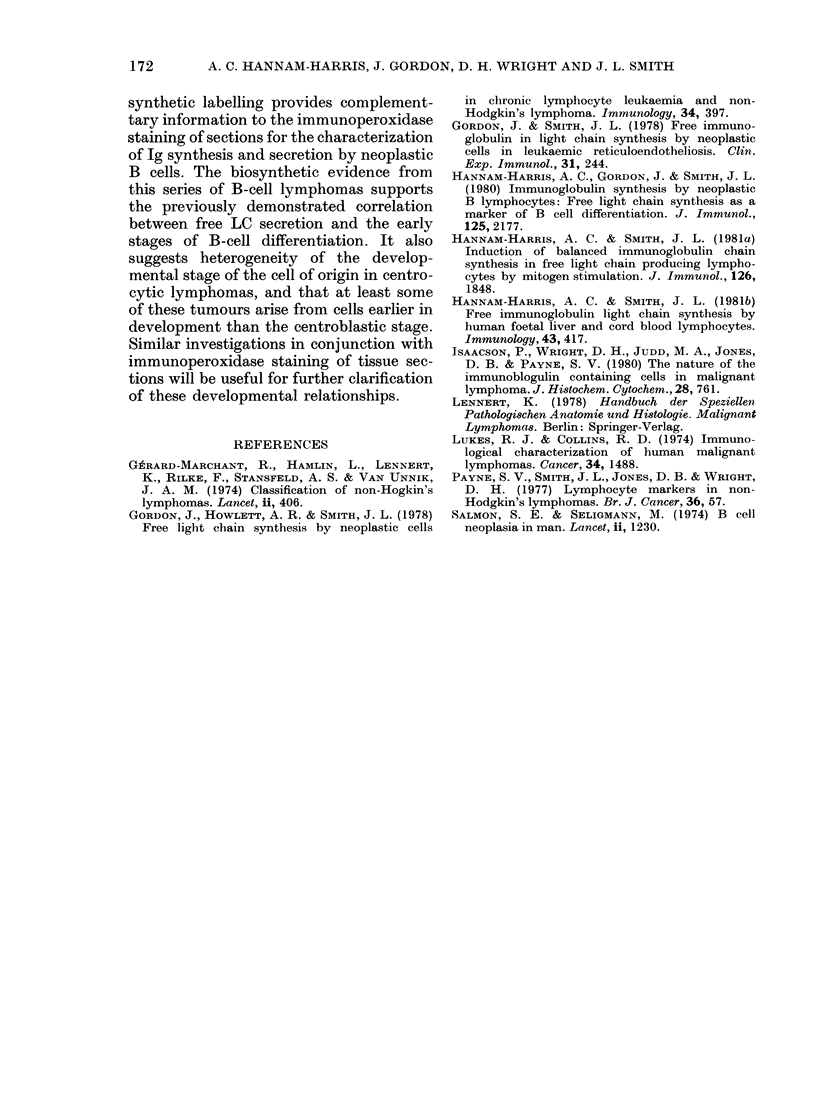# Correlation between Ig-synthesis patterns and lymphoma classification.

**DOI:** 10.1038/bjc.1982.180

**Published:** 1982-08

**Authors:** A. C. Hannam-Harris, J. Gordon, D. H. Wright, J. L. Smith

## Abstract

This study examines the link between free immunoglobulin (Ig) light-chain (LC) secretion and the developmental stage of the neoplastic B-cell of origin in B-cell lymphomas. The Kiel developmental scheme for lymphoma classification has been used to define the tumour-cell populations. Twenty-four B-cell lymphomas have been studied. In the small-lymphocytic lymphoma group, secreted Ig consisted of LC exclusively or in excess over heavy-chain (HC). Lymphomas of follicular-centre-cell origin, considered to be from cells further along the normal B-cell differentiation pathway, can be divided into centroblasts or centrocytes according to their histological appearance in tissue sections. Centroblastic lymphomas exhibited strong surface IgM or IgG expression, and the secretion of whole Ig was higher than by cells from the small-lymphocytic lymphomas. Synthesis of HC and LC was balanced in these cultures, both intracellularly and in secreted material. The centrocytic lymphomas comprised a functionally more heterogeneous group, the SIg varied in intensity and was of surface IgM, D or G. Likewise Ig synthesis was variable in quantity and composition, some cases secreting LC exclusively while others, including the cases expressing SIgG, secreted balanced HC and LC. In the Kiel classification centrocytes are considered to be more mature than centroblasts. Our data suggest that centrocytic lymphomas are heterogeneous in origin, some preceding and others following centroblasts in the B-cell maturation sequence. These data are discussed in relation to current concepts of B-cell maturation and lymphoma histology.


*Br. J. Cancer* (1982) **46**, 167

### CORRELATION BETWEEN Ig-SYNTHESIS PATTERNS AND LYMPHOMA CLASSIFICATION

A. C. HANNAM-HARRIS, J. GORDON, D. H. WRIGHT AND J. L. SMITH

*From the Regional Immunology Service, c/o Tenovus Research Laboratory, Southampton General Hospital, Southampton, Hants SO9 4XY*

Received February 1982  Accepted 6 April 1982

**Summary.—This study examines the link between free immunoglobulin (Ig) light-chain (LC) secretion and the developmental stage of the neoplastic B-cell of origin in B-cell lymphomas. The Kiel developmental scheme for lymphoma classification has been used to define the tumour-cell populations. Twenty-four B-cell lymphomas have been studied. In the small-lymphocytic lymphoma group, secreted Ig consisted of LC exclusively or in excess over heavy-chain (HC). Lymphomas of follicular-centre-cell origin, considered to be from cells further along the normal B-cell differentiation pathway, can be divided into centroblasts or centrocytes according to their histological appearance in tissue sections. Centroblastic lymphomas exhibited strong surface IgM or IgG expression, and the secretion of whole Ig was higher than by cells from the small-lymphocytic lymphomas. Synthesis of HC and LC was balanced in these cultures, both intracellularly and in secreted material. The centrocytic lymphomas comprised a functionally more heterogeneous group, the SIg varied in intensity and was of surface IgM, D or G. Likewise Ig synthesis was variable in quantity and composition, some cases secreting LC exclusively while others, including the cases expressing SIgG, secreted balanced HC and LC. In the Kiel classification centrocytes are considered to be more mature than centroblasts. Our data suggest that centrocytic lymphomas are heterogeneous in origin, some preceding and others following centroblasts in the B-cell maturation sequence. These data are discussed in relation to current concepts of B-cell maturation and lymphoma histology.**

STUDIES INVESTIGATING the biosynthesis of immunoglobulin (Ig) by cells from B-cell CLL, B-cell lymphomas and leukaemic reticuloendotheliosis (Gordon *et al.*, 1978; Gordon & Smith, 1978; Hannam-Harris *et al.*, 1980) have noted differences in the Ig light chain (LC) and heavy chain (HC) composition of secreted Ig, related to the surface-marker characteristics of the tumour cells. Cells with surface Ig (SIg) staining patterns of the class and intensity normally associated with early B cells, have been shown to secrete small quantities of Ig consisting of LC exclusively or in large excess over combined Ig. In contrast, cells with SIg expression typical of B cells at later stages of development, synthesize and secrete larger quantities of Ig of balanced LC and HC composition.

The present study has investigated further this correlation between the stage of B-cell development and excess free LC secretion, within the B-cell lymphomas.

The lymphomas comprise a heterogeneous group of neoplasms displaying wide variation in cell morphology, lymph-node histology and prognosis. However, the realization that neoplastic cells in the tumours share properties with normal lymphocytes, and are considered to represent "frozen" stages in normal maturation (Salmon & Seligmann, 1974; Lukes & Collins, 1974), has promoted classification systems relating tumours to their cell of origin in a scheme of normal cell development within reactive lymph nodes.

The two most generally used developmental classifications are those of Lennert (1978) and Lukes & Collins (1974). Each proposes a scheme of normal lymphocyte development within the lymph node, and identifies lymphomas according to the normal counterpart of the predominant tumour-cell type, identified histologically. The schemes differ in terminology and some aspects of direction of transformation of cell types within follicle centres, but share a common conceptual approach to lymphoma classification. The terminology used in this study is that of the Kiel classification (Gérard-Marchant *et al.*, 1974).

Both the Kiel and Lukes and Collins classifications propose that within the line of B-cell development in lymph nodes, small lymphocytic cells, the lymph-node counterparts of chronic lymphocytic leukaemic cells, are at an early stage of development and occur outside the follicle centres. Cells of the follicle centre represent later stages of development, and are divided morphologically into two groups, the centroblasts (non-cleaved follicular-centre cells) and the centrocytes (cleaved follicular-centre cells). Further progression through immunoblastic transformation to plasmacytoid differentiation or memory cells, occurs outside the follicle centres.

This study comprises a series of 24 cases of B-cell lymphoma, grouped histologically according to the Kiel classification. The patterns of Ig synthesis by neoplastic cells in culture have been correlated with the position of the lymphoma in this classification, which has been used to define the relative maturity of the neoplastic cell of origin.

### MATERIALS AND METHODS

*Patients and cell preparation.*—Lymph-node or spleen biopsy material from 24 cases of B-cell lymphoma was investigated. All biopsies were shown to be involved, and classified histologically according to the Kiel scheme. Tumours were categorized as 'centrocyte predominant' if 70% or more of the cells showed the morphology of small centrocytes, and as 'centroblast predominant' if 30% or more of the cells were centroblasts. Most of the centrocyte-predominant tumours had a follicular structure, whereas those with centroblast predominance were diffuse. Final preparations contained 30–90% neoplastic cells, as judged by marker analysis, with most containing >50% neoplastic cells, accounting for >90% of the SIg-staining cells. Tissue was minced through sterile wire mesh and the resultant cell suspension prepared over Ficoll-Triosil, as previously described (Payne *et al.*, 1977). Cells cellected at the interface were washed ×3 and in all cases were >90% viable by trypan-blue exclusion, remaining >80% viable after 18h culture.

*Cell receptors.*—The rosette test for identification of cells with receptors for sheep erythrocytes (E) has been described elsewhere (Payne *et al.*, 1977). Surface Ig was characterized by staining of cell suspension with fluorescein-conjugated antisera to Ig HC and LC. Cell smears fixed in methanol and washed in saline were also stained with fluorescein-conjugated antisera by the direct method for the detection of cIg. Controls were included in all experiments. The fluorescein-labelled preparations were examined using a Leitz Orthoplan microscope fitted with a HB200 mercury-vapour Ploem illuminator.

*Ig synthesis.*—The biosynthetic techniques and subsequent detection and characterization of labelled Ig have been described in detail previously (Gordon *et al.*, 1978).

Briefly, cells at $5 \times 10^6$/ml were incubated in leucine-free medium containing $^3$H-leucine at 25 $\mu$Ci/ml for 18 h at 37°C. Cells were separated from the supernatant by centrifugation (150 *g*, 15 min) and lysed in phosphate-buffered saline containing detergent NP40 (Nonidet P40, B.D.H., Poole) and proteolytic inhibitors. Both cell lysates and supernatants were spun (35,000 *g*, 30 min) to remove cell debris.

Labelled protein in the culture supernatant and cell lysate was estimated by precipitation with 10% trichloroacetic acid (TCA). Labelled Ig was precipitated using a double-layer antibody technique with sheep antiserum specific for human Ig as the first antibody, and rabbit antiserum with activity to sheep Ig

TABLE I.—*Ig expression and synthesis by neoplastic lymphocytes from non-Hodgkin's lymphoma*

| Patient and lymphomas classification | E rosetting (%) | SIg (%) | SIg class | SIg intensity | cIg (%) | Supernatant Ig ppt. by ā Kc | ā λc |
|---|---|---|---|---|---|---|---|
| Small lymphocytic | | | | | | | |
| COB | 26 | 40 | Mk | + | – | n.d. | n.d. |
| KIN | 1 | 50 | Mλ | + | – | <5 | 100 |
| COW | 2 | 90 | M(G)k | + | – | 95 | 5 |
| DOR | 26 | 46 | M(G)k | + | 10Mk | 100 | <5 |
| HER | 42 | 58 | MDk | + | – | 90 | 10 |
| GRE | 23 | 80 | MDk | ++ | – | 100 | 0 |
| RUM | 38 | 97 | MDk | +++ | – | 100 | <10 |
| Follicular-centre cell | | | | | | | |
| Centrotype predominant | | | | | | | |
| SMI | 11 | 53 | Mk | + | – | 100 | 0 |
| AND | 6 | 35 | MDλ | + | – | 0 | 100 |
| KNI | 70 | 60 | MDk | ++ | – | 95 | n.d. |
| NEW | 10 | 91 | MDk | +++ | – | 95 | <10 |
| BAM | 25 | 75 | M(G)k | ++ | – | 95 | 5 |
| MAC | 22 | 63 | Mλ | ++ | – | 0 | 100 |
| PEC | 40 | 70 | Mk | ++ | – | 80 | 20 |
| SEW | 5 | 85 | Mλ | +++ | – | 0 | 100 |
| CPR | 7 | 28 | Gλ | ++ | – | 0 | 100 |
| JSM | 20 | 22 | Gk | +++ | – | 100 | 0 |
| HAW | 11 | 46 | Gλ | +++ | – | n.d. | n.d. |
| Centroblast predominant | | | | | | | |
| FRI | 18 | 70 | Mλ | ++ | – | 0 | 100 |
| GLA | 27 | 50 | Mk | +++ | – | 100 | 0 |
| RIT | 28 | 56 | Mλ | +++ | – | 0 | 100 |
| TUR | 20 | 60 | Mk | ++ | 50 Mk | 100 | 0 |
| WAR | 28 | 71 | Mk | +++ | – | 100 | 0 |
| CAR | 65 | 30 | Gλ | +++ | 10 Gλ | 0 | 100 |

as second antibody. Normal sheep IgG was used as first antibody in control precipitations. The Ig precipitations were washed ×4 in cold PBS containing NP40 and cold leucine before counting for quantitation or preparation for gel analysis. Reduced and alkylated samples were analysed on 7·5% SDS-polyacrylamide-gel electrophoresis (SDS-PAGE) run concurrently with radioactive markers. Gels were sliced, solubilized and counted.

Labelled Ig LC and HC class was determined by precipitation with specific first antibody. The molar ratio of light chain to heavy chain (LC:HC) in labelled Ig was determined from the counts associated with peaks of LC and HC on SDS-PAGE. Free LC synthesis was confirmed by precipitation with antisera against free LC determinants.

### RESULTS

The series of cases of B-cell lymphoma were grouped according to histological classification in the Kiel scheme. The results of Ig expression and synthesis by these groups are presented in Tables I and II.

The neoplastic cells from all patients expressed SIg, the intensity ranging from very weak to strong. All cases synthesized and secreted detectable levels of Ig during the 18h culture. The LC specificity, determined by specific precipitation, was of a single class, corresponding to that on the cell-surface membrane. HC class in secreted material, identified by mobility on SDS-PAGE relative to myeloma markers, also corresponded with that on the cell surface, confirmed in two cases (DOR, MAC) by precipitation with class-specific antisera.

Neoplastic cells from the small-lymphocyte group stained weakly for SIg, predominantly of M, or M+D isotypes. Cytoplasmic Ig was demonstrable by fluorescent staining in only one case (DOR) where a lung biopsy contained 10% cells staining for cIgM k. The secretion of

TABLE II.—*Ig synthesis and secretion by cells from non-Hodgkin's lymphoma*

(sup. = supernatant; lys. = lysate)

| Patient and lymphoma classification | Ig (%TCA) | sup./lys. Ig | Combined Ig secretion sup./lys. Ig | LC:HC molar ratio sup. | lys. |
|---|---|---|---|---|---|
| Small lymphocytic | | | | | |
| COB | 3·9 | 4·9 | 0 | LC only | 11·4 |
| KIN | 1·9 | 1·1 | 0 | LC only | 3·5 |
| COW | 0·4 | 1·0 | 0 | LC only | 11·0 |
| DOR | 2·4 | 1·7 | 1·1 | 2·8 | 0·0 |
| HER | 0·4 | 0·9 | 0 | LC only | 1·8 |
| GRE | 2·2 | 0·9 | 0·6 | 2·6 | 1·1 |
| RUM | 1·5 | 1·2 | 0·6 | 4·5 | 1·8 |
| FCC | | | | | |
| CC predominant | | | | | |
| SMI | 1·1 | 1·1 | 0 | LC only | 7·5 |
| AND | 0·3 | 0·4 | 0 | LC only | 2·5 |
| KNI | 0·6 | 0·6 | 0·4 | 2·2 | 1·1 |
| NEW | 0·9 | 0·5 | 0·1 | 12·0 | 1·2 |
| BAM | 3·1 | 0·3 | 0·2 | 2·6 | 1·0 |
| MAC | 4·1 | 0·7 | 0·7 | 1·0 | 1·0 |
| PEC | 0·2 | 1·0 | 0·8 | 2·0 | 0·0 |
| SEW | 12·0 | 2·0 | 0 | LC only | 1·0 |
| CPR | 0·4 | 1·0 | 0·8 | 2·0 | 1·2 |
| JSM | 1·4 | 0·2 | 0·2 | 1·8 | 1·2 |
| HAW | 1·3 | 0·7 | 0·7 | 1·1 | 0·7 |
| CB predominant | | | | | |
| FRI | 0·5 | 2·8 | 2·8 | 1·0 | 1·0 |
| GLA | 7·6 | 1·6 | 1·6 | 0·9 | 1·0 |
| RIT | 4·8 | 1·9 | 1·8 | 1·2 | 1·0 |
| TUR | n.d. | n.d. | n.d. | 1·1 | 0·6 |
| WAR | 0·9 | 2·4 | 2·3 | 1·2 | 1·2 |
| CAR | 2·6 | 1·0 | 1·0 | 1·1 | 1·1 |

whole Ig, indicated by the ratio of combined Ig in the culture medium to that detected intracellularly, was low or absent. All cases, however, secreted free LC, and in 4 (COB, KIN, COW, HER) LC was the only Ig product detectable in the culture supernatant. The lysate LC:HC synthetic imbalance, though present, was less marked.

The centroblastic lymphomas demonstrated strong SIg expression of M or G class, and in two cases (TUR, CAR) cIg was detectable by fluorescent staining. This group showed a higher ratio of secreted to intracellular labelled Ig than the lymphocytic category, and LC:HC was balanced both intracellularly and in secreted material. The lymphomas with predominantly centrocytic histology comprised a functionally more heterogeneous group. Surface Ig staining was generally strong of M, G or M with D classes. No cIg was detected by fluorescent staining. The proportion of secreted to intracellular Ig and LC and HC composition, varied from secretion of LC exclusively in some cases, to balanced secretion in others. Intracellularly, synthetic imbalance were less marked.

### DISCUSSION

Previous investigations (Gordon *et al.*, 1978; Hannam-Harris *et al.*, 1980) have reported a strong, consistent correlation between the class and intensity of SIg expression, and the synthesis and secretion of free LC by neoplastic cells in culture. Free LC synthesis has been noted similarly in subpopulations of normal adult spleen cells and in cultures of fetal liver lymphocytes (Hannam-Harris & Smith, 1981*a*, *b*) and appears to be associated with early stages of B-cell development in normal and neoplastic tissue. The application of these findings to the elucidation of the sequence of cell development within follicle centres has been explored with reference to current developmental schemes for lymphoma classification.

In all biopsies examined in this study the Leishman staining of slide preparations of extracted cells showed a close relationship between the morphology of the cells in suspension and that identified histologically in sections of the solid tumour. Immunological markers on extracted cells demonstrated monotypic SIg expression and precipitated labelled Ig was of a single LC class, indicating minimal contribution from uninvolved cells to the synthesis patterns observed.

Cells from small-lymphocytic lymphomas had weak staining for SIg of M or M + D classes and, with a single exception (DOR), failed to stain for cIg. This pattern of Ig expression is compatible with normal B cells early in development (Salmon & Seligmann, 1974). In culture, these cells secreted little or no labelled whole Ig, providing additional evidence for a cell of origin early on the B-differentiation pathway. Secreted Ig consisted of LC exclusively or in large excess over HC. LC and HC were more balanced intracellularly, as generally noted in LC-secreting cells (Hannam-Harris *et al.*, 1980). The exceptional case (DOR) where synthetic imbalance was small, demonstrated small-lymphocytic histology but was atypical in its presentation, with lung involvement and plasmacytoid differentiation.

Lymphomas of follicular-centre-cell origin, which are considered to arise from cells further along the normal B-cell differentiation pathway than small-lymphocytic tumours, were divided into centroblastic and centrocytic groups, according to the predominant cell type identified histologically in tissue sections. Cells from the predominantly centroblastic lymphomas exhibited strong surface expression of IgM or IgG, and cIg was demonstrated in two cases. Secretion of whole Ig was higher than that found in the small-lymphocytic lymphomas, and is characteristic of cells further along the differentiation pathway to mature Ig secreting cells. Synthesis of HC and LC was balanced in these cultures both intracellularly and in secreted material. In contrast, the centrocytic lymphomas comprised a functionally more heterogeneous group. Surface Ig expression varied in intensity but was generally stronger than in the small-lymphocytic lymphomas, and was IgG in three cases. Likewise, Ig synthesis was variable in quantity and composition, some cases secreting LC exclusively, whilst others, including the cases expressing SIgG, secreted more balanced HC and LC.

We have attempted to relate these observations to the various schemes for the development of follicular centre cells. Lukes & Collins (1974) propose that small lymphocytes entering the follicle progress through small and large centrocytes to centroblasts, with further maturation to immunoblasts and plasma cells outside the follicle. The B-cell maturation sequence proposed by Lennert (1978) differs significantly in the developmental progression within follicles, and shows centroblasts giving rise to centrocytes. Our finding that some tumours within the centrocyte-predominant group secrete LC exclusively suggests a developmental proximity of some of these tumours to small-lymphocytic lymphomas at an early stage in B-cell maturation. However, other tumours in the centrocytic group showed Ig synthesis patterns indistinguishable from those of the centroblastic group. Furthermore, cIg has been demonstrated within reactive and neoplastic centrocytes (Isaacson *et al.*, 1980). The centrocyte-predominant tumours thus appear to be a heterogeneous group, supporting the concept that transformation between centroblasts and centrocytes may occur in more than one direction. In this sense these findings are at variance with both developmental classifications, which show a unidirectional differentiation of centrocytes to centroblasts or *vice versa*.

Further analysis of the developmental relationship between follicular-centre cells using these techniques is indicated. Biosynthetic labelling provides complementtary information to the immunoperoxidase staining of sections for the characterization of Ig synthesis and secretion by neoplastic B cells. The biosynthetic evidence from this series of B-cell lymphomas supports the previously demonstrated correlation between free LC secretion and the early stages of B-cell differentiation. It also suggests heterogeneity of the developmental stage of the cell of origin in centrocytic lymphomas, and that at least some of these tumours arise from cells earlier in development than the centroblastic stage. Similar investigations in conjunction with immunoperoxidase staining of tissue sections will be useful for further clarification of these developmental relationships.

### REFERENCES

GÉRARD-MARCHANT, R., HAMLIN, L., LENNERT, K., RILKE, F., STANSFELD, A. S. & VAN UNNIK, J. A. M. (1974) Classification of non-Hogkin's lymphomas. *Lancet*, **ii**, 406.

GORDON, J., HOWLETT, A. R. & SMITH, J. L. (1978) Free light chain synthesis by neoplastic cells in chronic lymphocyte leukaemia and non-Hodgkin's lymphoma. *Immunology*, **34**, 397.

GORDON, J. & SMITH, J. L. (1978) Free immunoglobulin in light chain synthesis by neoplastic cells in leukaemic reticuloendotheliosis. *Clin. Exp. Immunol.*, **31**, 244.

HANNAM-HARRIS, A. C., GORDON, J. & SMITH, J. L. (1980) Immunoglobulin synthesis by neoplastic B lymphocytes: Free light chain synthesis as a marker of B cell differentiation. *J. Immunol.*, **125**, 2177.

HANNAM-HARRIS, A. C. & SMITH, J. L. (1981*a*) Induction of balanced immunoglobulin chain synthesis in free light chain producing lymphocytes by mitogen stimulation. *J. Immunol.*, **126**, 1848.

HANNAM-HARRIS, A. C. & SMITH, J. L. (1981*b*) Free immunoglobulin light chain synthesis by human foetal liver and cord blood lymphocytes. *Immunology*, **43**, 417.

ISAACSON, P., WRIGHT, D. H., JUDD, M. A., JONES, D. B. & PAYNE, S. V. (1980) The nature of the immunoblogulin containing cells in malignant lymphoma. *J. Histochem. Cytochem.*, **28**, 761.

LENNERT, K. (1978) *Handbuch der Speziellen Pathologischen Anatomie und Histologie. Malignant Lymphomas*. Berlin: Springer-Verlag.

LUKES, R. J. & COLLINS, R. D. (1974) Immunological characterization of human malignant lymphomas. *Cancer*, **34**, 1488.

PAYNE, S. V., SMITH, J. L., JONES, D. B. & WRIGHT, D. H. (1977) Lymphocyte markers in non-Hodgkin's lymphomas. *Br. J. Cancer*, **36**, 57.

SALMON, S. E. & SELIGMANN, M. (1974) B cell neoplasia in man. *Lancet*, **ii**, 1230.